# Synthesis of FeO@SiO_2_–DNA core–shell engineered nanostructures for rapid adsorption of heavy metals in aqueous solutions†

**DOI:** 10.1039/d0ra06743a

**Published:** 2020-10-27

**Authors:** David Patiño-Ruiz, Lars Rehmann, Mehrab Mehrvar, Edgar Quiñones-Bolaños, Adriana Herrera

**Affiliations:** Engineering Doctorate Program, Nanomaterials and Computer-Aided Process Engineering Research Group, Universidad de Cartagena Cartagena 130010 Colombia; Department of Chemical and Biochemical Engineering, University of Western Ontario London N6A 3K7 Canada; Department of Chemical Engineering, Ryerson University Toronto M5B 2K3 Canada; Civil Engineering Program, Environmental Modelling Research Group, Universidad de Cartagena Cartagena 130010 Colombia; Chemical Engineering Program, Nanomaterials and Computer-Aided Process Engineering Research Group, Universidad de Cartagena Cartagena 130010 Colombia aherrerab2@unicartagena.edu.co

## Abstract

Creating novel and innovative nanostructures is a challenge, aiming to discover nanomaterials with promising properties for environmental remediation. In this study, the physicochemical and adsorption properties of a heterogeneous nanostructure are evaluated for the rapid removal of heavy metal ions from aqueous solutions. Core–shell nanostructures are prepared using iron oxide cores and silica dioxide shells. The core is synthesized *via* the co-precipitation method and modified *in situ* with citric acid to grow a carboxyl layer. The shell was hydrolyzed/condensed and then functionalized with amine groups for ds-DNA condensation *via* electrostatic interaction. The characterization techniques revealed functional FeO@SiO_2_–DNA nanostructures with good crystallinity and superparamagnetic response (31.5 emu g^−1^). The predominant superparamagnetic nature is attributed to the citric acid coating. This improves the dispersion and stability of the magnetic cores through the reduction of the dipolar–dipolar interaction and the enhancement of the spin coordination. The rapid adsorption mechanism of FeO@SiO_2_–DNA was evaluated through the removal of Pb(ii), As(iii), and Hg(ii). A rapid adsorption rate is observed in the first 15 min, attributed to a heterogeneous chemisorption mechanism based on electrostatic interactions. FeO@SiO_2_–DNA shows higher adsorption efficiency of 69% for Pb(ii) removal compared to As(iii) (51%) and Hg(ii) (41%). The selectivity towards Pb(ii) is attributed to the similar acid nature to ds-DNA, where the ionic strength interaction provides good affinity and stability. The facile synthesis and rapid adsorption suggest a promising nanostructure for the remediation of water sources contaminated with heavy metal ions and can be extended to other complex molecules.

## Abstract 2

Facile synthesis of well-dispersed and magnetic FeO@SiO_2_–DNA nanostructures with electrostatic active sites for interaction and rapid adsorption of heavy metals.

## Graphical abstract

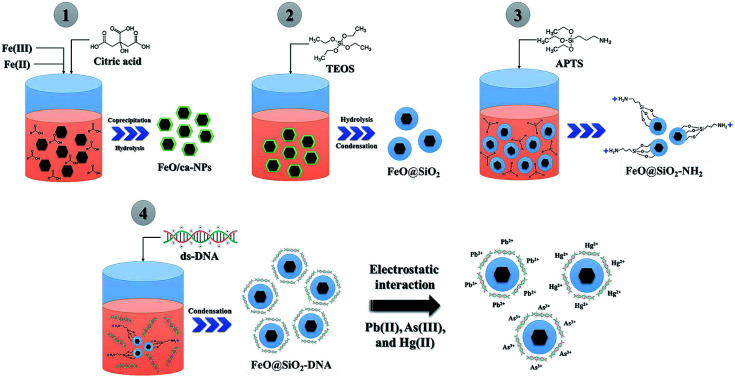

## 1. Introduction

Urbanization and industrialization have increased water source contamination due to the indiscriminate release of heavy metal ions into the environment.^1^ Globally, heavy metal ions are mainly introduced into different ecosystems through anthropogenic activities.^2^ Such activities can include mining, smelting,^3^ chemicals and petrochemical manufacturing,^4^ energy generation,^1^ domestic and industrial waste discharges,^5^ agricultural wastes,^6^ metallurgy,^7^ and construction.^8^ The most toxic and hazardous heavy metal ions in water include As(iii), As(v), Cr(iii), Cr(vi), Cu(ii), Hg(ii), Pb(ii), Cd(ii), Ni(ii), Zn(ii),^9^ and Mn(ii).^10^ These metal ions are toxic, stable, non-degradable, and can easily accumulate in ecosystems and living organisms.^6^ The prolonged exposure becomes harmful to plants, animals, and humans, causing severe poisoning, diseases, and mutations.^1^ This exposure increases the chance of suffering cancer, nausea, mental disorders, liver and kidney failures,^2^ bone deformation,^11^ lethargy, hypertension, and depression.^3^

Considering the high stability of heavy metal ions in water sources, the allowable limits established by the World Health Organization (WHO) can be easily exceeded.^12^ Some heavy metal ions can easily migrate, reaching groundwater sources and affecting the quality of drinking water. Groundwater can also function as a carrier of heavy metal ions increasing the impacts by contaminating soils.^13^ Different treatment techniques are being explored to control the high concentration in the water sources, which are reported statistical quantities of mt per year of Hg (0.015), Cu (3.4), Pb (5.0), Mn (15.0), and Ni (1.0).^14^ Techniques such as oxidation, sedimentation, coagulation, ion exchange, reverse osmosis,^4^ photodegradation, precipitation, flocculation, and membrane filtration,^15^ have been investigated. Currently, adsorption has been proven as an efficient route to remove heavy metal ions from groundwater. This method is considered as low cost, efficient,^14^ simple in design, and facile in handling.^16^ Moreover, the use of advanced and novel organic and inorganic adsorbents improves the adsorption viability and sustainability for groundwater treatments.^17^

Nanomaterials are suitable adsorbents for the removal of heavy metal ions from water sources. The unique physiochemical properties at nanoscale provide higher efficiency, versatility, and adsorption rates compared to other bulk adsorbents.^18^ Adsorbents such as core–shell nanostructures are the combination of two or more types of materials, which allow improving the mechanical, chemical, and physical properties. Iron oxide nanoparticles (FeO-NPs) are frequently used as the core due to the exceptional superparamagnetic property, providing the facility to control, recover, and regenerate the adsorbent nanostructure.^19^ However, FeO-NPs possess some limitations such as hydrophobic surface, easy chemical degradation in acidic conditions, and the formation of aggregates due to strong dipolar–dipolar interaction.^20^ To overcome these limitations, functionalization of the surface, promotes an enhancement in the stability, compatibility, functionality,^21^ and reactivity of the FeO-NPs.^22^ Among the functional materials, compounds containing carboxyl (–COOH) groups can promote an improvement in the dispersion in water, total surface area, and chemical resistance.^23^

The growth of silica (SiO_2_) shell on the magnetic core provides direct protection, as well as confers more advantages such as high surface area, non-toxic behavior, compatibility, affinity, and stability.^24^ The SiO_2_ shell also improves the hydrophilic nature of the nanostructure, as an important factor for the adsorption of pollutants in water sources. The magnetic core can be easily coated with a SiO_2_ shell (FeO@SiO_2_) using a facile and inexpensive Stöber method.^25^ The presence of silanol (Si–OH) and siloxane (Si–O–Si) groups on the SiO_2_ shell promote few agglomerates of FeO@SiO_2_ due to the formation of hydrogen bonds.^26^ However, these groups also provide a large number of active sites for further functionalization with other molecules, and even for an improved adsorption process.^27^ Among those molecules, the presence of amine (–NH_2_) groups increases the massive interaction between the FeO@SiO_2_ with specific targets such as DNA chains,^28^ heavy metal ions,^27^ and organic pollutants.^29^ The enhanced surface promotes stability and good dispersion of the FeO@SiO_2_ by breaking the hydrogen bonds.

The amine-functionalized magnetic core–shell silica (FeO@SiO_2_–NH_2_) nanostructures can be modified with more complex molecules, aiming to achieve superior adsorption properties. Double-stranded DNA (ds-DNA) chains are a suitable alternative due to the formation of duplexes between the nucleobases (thymidine, thymine, and cytosine) and heavy metals such as Ag(ii), Hg(ii), and Pb(ii).^30^ The modification of FeO@SiO_2_–NH_2_ with ds-DNA can be carried out using a condensation strategy based on electrostatic interaction,^31^ which allows the compaction of the ds-DNA on to core–shell nanostructure (FeO@SiO_2_–DNA). Moreover, the negatively charged phosphate chains also provide a large number of bindings sites *via* electrostatic interaction for enhanced stability,^32^ selectivity, and adsorption efficiency.^33^ To the authors' best knowledge, most of the reported nanostructures containing DNA chains on the surface are used as biosensors for the detection of heavy metal ions rather than adsorption and removal.^32,34,35^ There is information in the open literature about the synthesis of similar core–shell nanostructures including aptamer-modified SiO_2_@Au,^36^ DNA/poly-l-methionine–gold,^37^ AuNPs–DNA conjugates,^38^ and DNA-functionalized graphene.^39^

The main objective of this study is the facile and novel synthesis method of FeO@SiO_2_–DNA combining various materials in one approach. The core consisted of FeO-NPs that were modified *in situ* with citric acid (FeO/ca-NPs). The morphological information showed a more dispersed FeO-NPs due to the citric acid coating. The FeO/ca-NPs were then coated by growing a SiO_2_ shell *via* the Stöber method. The FeO/ca-NPs allowed the formation of uniform core–shell (FeO@SiO_2_) nanostructures with homogeneous spherical shape and shell thickness. Further functionalization of FeO@SiO_2_ with –NH_2_ groups, provided a large number of active sites for condensation of ds-DNA chains onto the surface. The highly functional FeO@SiO_2_–DNA showed good crystallinity and superparamagnetic response, even after being functionalized with citric acid, SiO_2_, –NH_2_, and ds-DNA. Furthermore, adsorption experiments were performed to establish the adsorption efficiency of FeO@SiO_2_–DNA for the removal of Pb(ii), As(iii), and Hg(ii). The rapid adsorption mechanism was ascribed as chemisorption *via* electrostatic interaction between the nucleobases of the ds-DNA, and the cationic nature of the metal ions. Additionally, the heavy metal ions showed high stability on the FeO@SiO_2_–DNA after being immersed into the eluent agents, suggesting the FeO@SiO_2_–DNA as suitable nano-adsorbent for the treatment of water sources, including other heavy metal ions.

## 2. Experimental methods

### 2.1. Materials

Iron(iii) chloride hexahydrate (FeCl_3_·6H_2_O, 97%), iron(ii) chloride tetrahydrate (FeCl_2_·4H_2_O, 99%), sodium hydroxide (NaOH, 98%), citric acid (ca, 99.5%), tetraethyl orthosilicate (TEOS, 98%), ammonium hydroxide (NH_4_OH, 28–30%), (3-aminopropyl)triethoxysilane (APTES, 98%), and acetic acid (CH_3_CO_2_H, 99.7%) were acquired from SigmaAldrich® for preparation and modification of the nanostructures. Double-stranded deoxyribonucleic acid (ds-DNA) sodium salt from salmon testes, tris hydrochloride (Tris–HCl, 99%), ethylenediaminetetraacetic acid (EDTA, 99%), sodium chloride (NaCl, 99.5%), and polyethylene glycol 8000 (PEG-8000) were also purchased from SigmaAldrich® for condensation of DNA onto the core–shell surface. Lead(ii) nitrate (Pb(NO_3_)_2_, 99.9%), sodium (meta)arsenite (NaAsO_2_, 90%), and mercury chloride (HgCl_2_, 99.5%) were obtained from SigmaAldrich® for the adsorption experiments. Ethanol absolute, reagent alcohol, and distilled water were used in all experiments.

### 2.2. FeO-NPs and FeO/ac-NPs synthesis

FeO-NPs were prepared according to the traditional co-precipitation method. Initially, FeCl_3_·6H_2_O and FeCl_2_·4H_2_O solutions (50 mL each) were prepared considering a 2 : 1 molar ratio, respectively. The solutions were mixed into a three-neck flask with 120 rpm of mechanical stirring and heated up to 80 °C. Once the temperature was reached, NaOH solution (1 M) in distilled water (100 mL) was added dropwise and left to react for 30 min. Surface modification of FeO-NPs with –COOH functional groups was carried out *in situ*, by adding citric acid (0.5 g in 50 mL of distilled water) solution, leaving to react for 30 min under the same conditions. After the reaction time was completed, the solution was cooled down at room temperature, and the black precipitate was then collected centrifuging at 20 000*g* force at 25 °C for 20 min. Centrifugation was repeated four times, after washing with distilled water (three times) and reagent alcohol (once). Finally, FeO/ac-NPs were dried in an oven at 70 °C overnight.

### 2.3. Growth of the SiO_2_ shell on the FeO/ac-NPs

The SiO_2_ shell was grown through the Stöber method based on a hydrolysis/condensation mechanism with the FeO/ac-NPs *in situ*. Hence, FeO/ac-NPs (75 mg) was added in distilled water (32 mL) and dispersed using a sonicator tip controlling the temperature for 10 min. Ethanol absolute (50 mL) was added into the solution and sonicated again for 20 min. After sonication, the solution was immediately placed on a magnetic stirrer under vigorous agitation (1000 rpm) at room temperature. TEOS (1.2 mL) precursor was added followed by NH_4_OH (4 mL), dropwise both. The solution was left to react for 24 h under vigorous stirring at room temperature. The as-synthesized FeO@SiO_2_ were collected by centrifuging at 20 000*g* force at 25 °C for 20 min. For purification, the FeO@SiO_2_ were re-dispersed in distilled water and then reagent alcohol two times each followed by magnetic decantation, intending to remove impurities, un-reactants, and single SiO_2_ nanoparticles that could have grown during the reaction. Finally, the FeO@SiO_2_ were dried in an oven at 70 °C overnight.

### 2.4. Preparation of FeO@SiO_2_–NH_2_

The surface of the FeO@SiO_2_ were functionalized using –NH_2_ groups. Here, FeO@SiO_2_ (30 mg) were added in ethanol absolute (60 mL) and dispersed by using a sonicator tip for 30 min. The dispersion was placed on a stirrer with vigorous agitation (1000 rpm), and a solution of APTES (200 μL) in ethanol absolute (2.3 mL) was added dropwise. The pH was adjusted to 8 using NH_4_OH (6.5 M). The solution was heated up to 50 °C and left to react for 24 h. The resulting FeO@SiO_2_–NH_2_ were collected with centrifugation at 20 000*g* force at 25 °C for 20 min. To remove unreacted APTES, the FeO@SiO_2_–NH_2_ were washed and centrifuge several times with distilled water and once with reagent alcohol. The FeO@SiO_2_–NH_2_ were dried in an oven 60 °C overnight for further condensation of ds-DNA.

### 2.5. Condensation of ds-DNA on FeO@SiO_2_–NH_2_

Condensation of ds-DNA chains onto the surface of FeO@SiO_2_–NH_2_ nanostructures were performed by electrostatic interaction. All the instrumentation used in this stage was previously autoclaved for sterilization, and the experiments were carried out into a laminar flow cabinet. Initially, a solution of FeO@SiO_2_–NH_2_ (10 mg mL^−1^) was prepared in distilled water followed by sonication for 10 min. The ds-DNA was dissolved in a TE buffer solution (100 μg mL^−1^), which was previously prepared using Tris–HCl (20 mM) and EDTA (2 mM). The ds-DNA (300 μL) solution was added in a PEG/NaCl (7.5%/1 M) mixture (300 μL), and then incubated in a shaker with 100 rpm at room temperature for 1 min. Next, a freshly prepared FeO@SiO_2_–NH_2_ dispersion (15 μL) was added to the as-incubated ds-DNA solution, leaving to incubate for 5 min under the same conditions. The as-prepared FeO@SiO_2_–DNA were magnetically separated, and the supernatant was then used to quantify the remaining ds-DNA in the solution using a microplate reader. The ds-DNA adsorption efficiency on the FeO@SiO_2_ was calculated according to eqn (1). Finally, the FeO@SiO_2_–DNA were washed several times with an ethanol absolute (70% v/v) solution to remove the excess of ds-DNA, dried at room temperature for 24 h, and stored at 4 °C.1
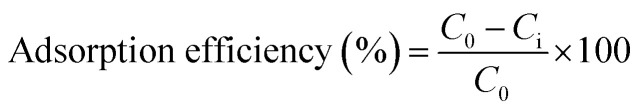
where *C*_0_, and *C*_i_ are the initial and final concentrations of ds-DNA in the supernatant (mg L^−1^).

### 2.6. Characterizations

Scanning electron microscopy (SEM) images were acquired from an LEO (Zeiss) 1540X with a beam operating between 3 and 30 kV. The sample was coated with osmium previous to SEM. The elemental composition was performed using the energy-dispersive X-ray (EDX) spectroscopy, in which the detector was coupled to the SEM. Transmission electron microscopy (TEM) images were collected on a Philips CM10 with an acceleration voltage of 100 kV. An available ImageJ Software was used to obtain the particle size histograms of nanoparticles and nanostructures. Brunauer–Emmett–Teller (BET) surface area analysis and Barrett–Joyner–Halenda (BJH) pore size and volume analysis were performed using a Gemini V2.00 from Micromeritics®. The ds-DNA quantification was determined in a Two Teacen M1000 microplate reader. X-ray diffraction (XRD) was performed using a Rigaku SmartLab X-ray diffraction system with Ni-filtered CuKα radiation (*λ* = 1.54059 Å) in Bragg–Brentano geometry, with a step-size of 0.02° in the range of 10 to 100°. A vibrating sample magnetometer (VSM) Versalab from Quantum Design, was used to collect the magnetization curves at 300 K between −30 kOe and 30 kOe. Functional groups were identified through Fourier transform infrared (FTIR) spectroscopy using an FTIR Vertex 70 from Bruker, equipped with an MCT detector. Total mercury(Hg) was determined by Direct Mercury Analysis (DMA) using a Dual cell DMA-80 Milestone, with detection and report limits of 0.07 and 0.22 ng, respectively. Inductively coupled plasma-mass spectrometry (ICP-MS) was performed for quantification of lead (using a He gas flow) and arsenic (no gas) in an Agilent 7700x Series equipment.

### 2.7. Heavy metals adsorption experiments and kinetics

Batch adsorption experiments were performed by triplicate using three different heavy metal ions (Pb(ii), As(iii), and Hg(ii)) solutions, to determine the adsorption efficiency of FeO@SiO_2_–DNA. Before adsorption experiments, the instrumentation and distilled water were autoclaved for sterilization. Stock solutions (10 ppm) of each heavy metal ion were prepared in distilled/sterilized water. FeO@SiO_2_–DNA (40 μg mL^−1^) were added into the heavy metal ion (50 mL) solution and then sonicated for 2 min for dispersion. All the experiments were carried out with a neutral pH. The solutions were placed in a shaker with a dark environment and constant agitation of 120 rpm at 22 °C for 24 h. After 24 h, the FeO@SiO_2_–DNA were magnetically separated, and the liquid samples were stored in amber vials for further performing of ICP-MS and DMA techniques. The samples containing Pb(ii) and As(iii) were preserved adding trace level nitric acid (0.5 mL) to adjust pH (<2). For Hg(ii), a trace grade HCl (0.5% HCl) amount (0.2 mL) was added. Accordingly, the adsorption capacity and adsorption efficiency were calculated by using eqn (2) and (3), respectively.2
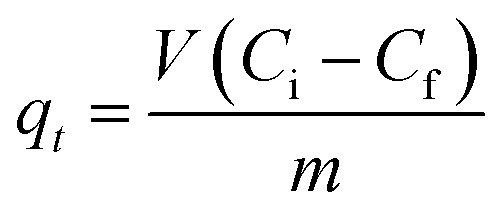
3
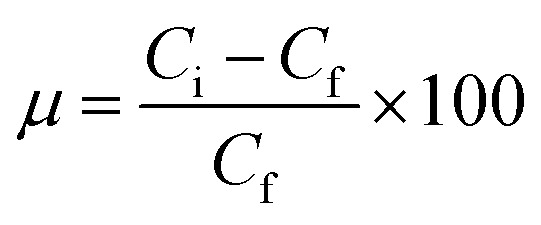
where *q*_*t*_ is the adsorption capacity (mg g^−1^), *μ* is the removal percentage, *V* is the solution volume (L), *C*_i_ and *C*_f_ are the initial and final concentration of heavy metal ions (mg L^−1^), respectively, and *m* is the mass of the FeO@SiO_2_–DNA (g).

### 2.8. Kinetic study

The adsorption kinetics were carried out by adding FeO@SiO_2_–DNA (40 μg mL^−1^) into the stock solution of heavy metal ions (50 mL) followed by sonication for 2 min. The solutions were placed in a shaker with a dark environment and constant agitation of 120 rpm at 22 °C for a total time of 120 min. Sample samples were taken out at 5, 10, 15, 30, 45, 60, and 120 min. Once the adsorption experiment was completed, the FeO@SiO_2_–DNA were separated using a magnet. Then, the liquid samples were stored in amber vials for further performing of ICP-MS and DMA techniques. Preservation of the samples was also performed to keep the heavy metal ions stabilized. The results were adjusted to four kinetic models to determine the adsorption mechanism of the FeO@SiO_2_–DNA. The kinetic models considered were pseudo-first order, pseudo-second order, Elovich, and intraparticle diffusion, which are accordingly expressed in eqn (4)–(7).^40^4
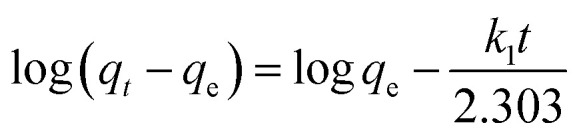
5
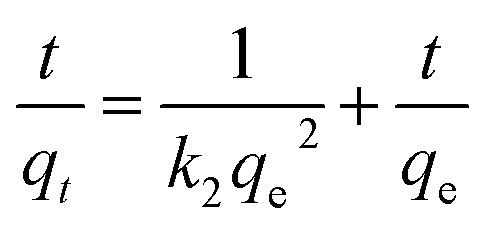
6
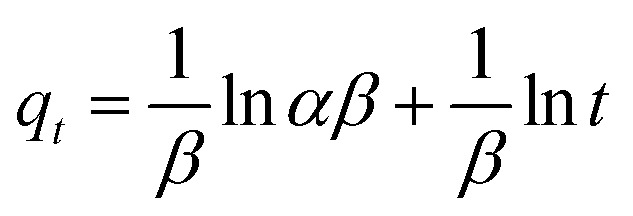

7 *q*_*t*_ = *k*_p_*t*^1/2^ + *C*

where *q*_*t*_ and *q*_e_ are the heavy metal ions adsorbed at the given and equilibrium time (mg g^−1^), respectively, *k*_1_ is the Pseudo-first order constant, *t* is the given time (min), *k*_2_ is the pseudo-second order constant, *α* and *β* are the Elovich adsorption and desorption rates, respectively, *C* is the boundary layer effect (mg g^−1^), and *k*_p_ is the intraparticle diffusion constant.

### 2.9. Desorption

Desorption experiments were performed to determine the stability and reusability of the FeO@SiO_2_–DNA. EDTA (0.05 mol L^−1^) solution was prepared for desorption of Pb(ii) and Hg(ii), and a NaOH (1 M) solution for desorption As(iii).^41–43^ The FeO@SiO_2_–DNA used for the adsorption experiments were re-dispersed in distilled water to remove the excess of heavy metal ions and then separated magnetically. The FeO@SiO_2_–DNA (40 μg mL^−1^) were added into the EDTA or NaOH solutions (50 mL). The batch system was placed in a shaker under a dark environment and constant agitation of 120 rpm at 22 °C for 24 h. Finally, the FeO@SiO_2_–DNA were magnetically separated, and the supernatant was stored in amber vials and preserved for further analysis. These desorption experiments were carried out by triplicate.

## 3. Results and discussion

### 3.1. Nanostructures synthesis and characterization

**Fig. 1:**
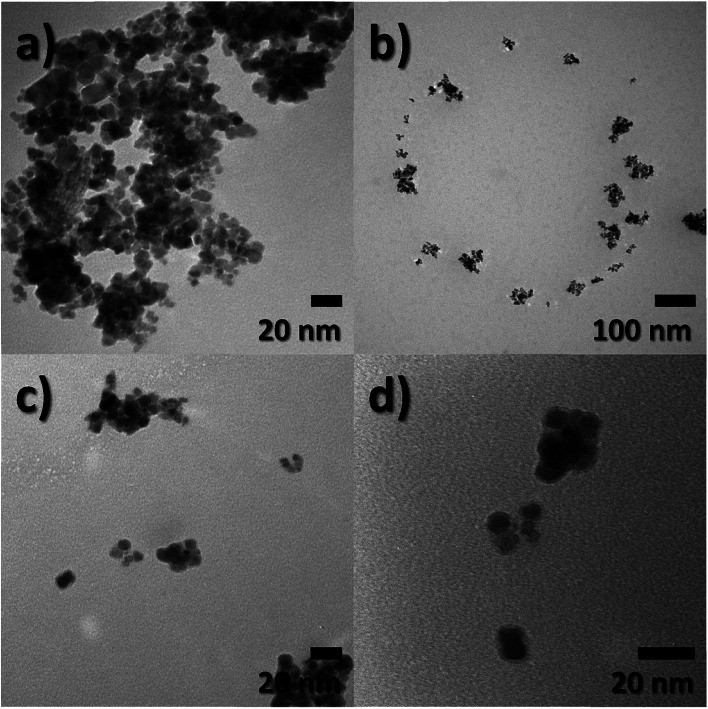
Transmission electron microscopy (TEM) images of (a) FeO-NPs and (b–d) FeO/ca-NPs.

Particle size, morphology, and structural information of FeO-NPs and FeO/ca-NPs were displayed in the TEM images of Fig. 1a–d. Fig. 1a shows a dense agglomeration which is a common characteristic due to the use of the co-precipitation method. The FeO-NPs possess a semi-spherical shape with an average diameter size of 10.2 ± 3.7 nm (see ESI, Fig. S1a†). Fig. 1b–d shows an increase in the nanoparticles' dispersion. The agglomeration was reduced due to the decrease in the dipolar–dipolar interaction between nanoparticles, which was promoted by the citric acid coating of FeO-NPs with a layer thickness of 2.2 ± 0.2 nm.^44^ Although some agglomerates are observed, the nanoparticles can be easily dispersed due to the strong affinity of the carboxylic acid with the aqueous mediums. The hydrophilic nature of the carboxylic acid promotes a hydration effect in the water, leading the stabilization and good dispersion of FeO/ca-NPs.^23^

**Fig. 2:**
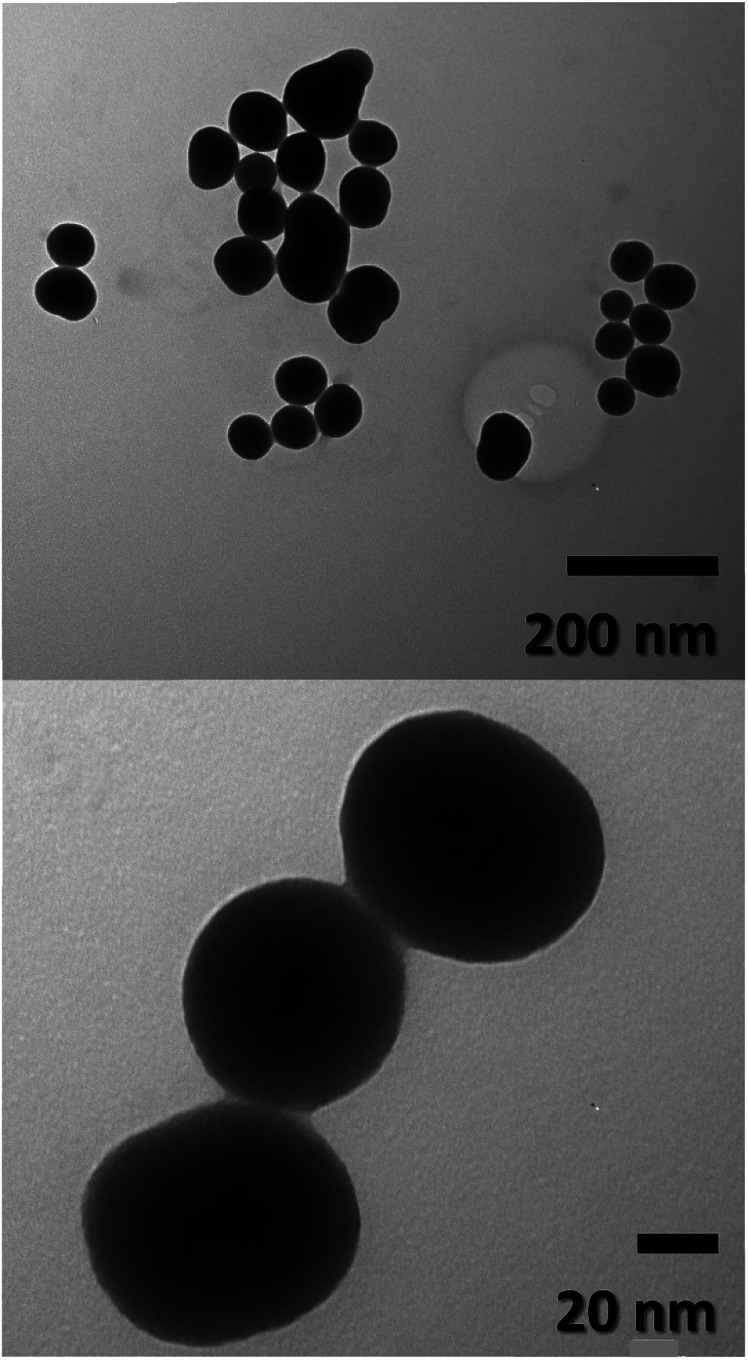
TEM images of FeO@SiO_2_.

TEM images of the FeO@SiO_2_ morphology are displayed in Fig. 2. The structure exhibits the core–shell shape using the combination of two different materials. Single magnetic cores can be observed, including small agglomerates forming the core. The presence of a single core suggests high dispersion and stability of FeO/ca-NPs after citric acid coating. Here, the citric acid coating promoted a more stable hydrolysis/condensation process of hydroxyl (OH^−^) groups from the carboxylic acid structure, resulting in controlled hydrolysis of TEOS.^45^ This controlled reaction allowed to synthesize single, uniform, and well-dispersed FeO@SiO_2_, rather than a bulk of nanoparticles coated with a SiO_2_ shell as reported in several studies.^26,27,45^ The FeO@SiO_2_ possess spherical and semi-spherical shapes with a wide distribution and average diameter size of 125 ± 27 nm (see ESI, Fig. S1b†). The increase in the diameter sizes of FeO@SiO_2_ was attributed to the small agglomerates forming the magnetic cores. Additionally, the SiO_2_ shell was uniform and homogenous due to the symmetric radius on the FeO/ca-NPs' surface, in which the thickness was calculated and the average was 38 ± 6 nm (see ESI, Fig. S1c†). The thickness is ascribed to a high hydrolysis/condensation ratio of TEOS, which is related to the number of available OH^−^ groups on the FeO/ca-NPs' surface.

**Fig. 3:**
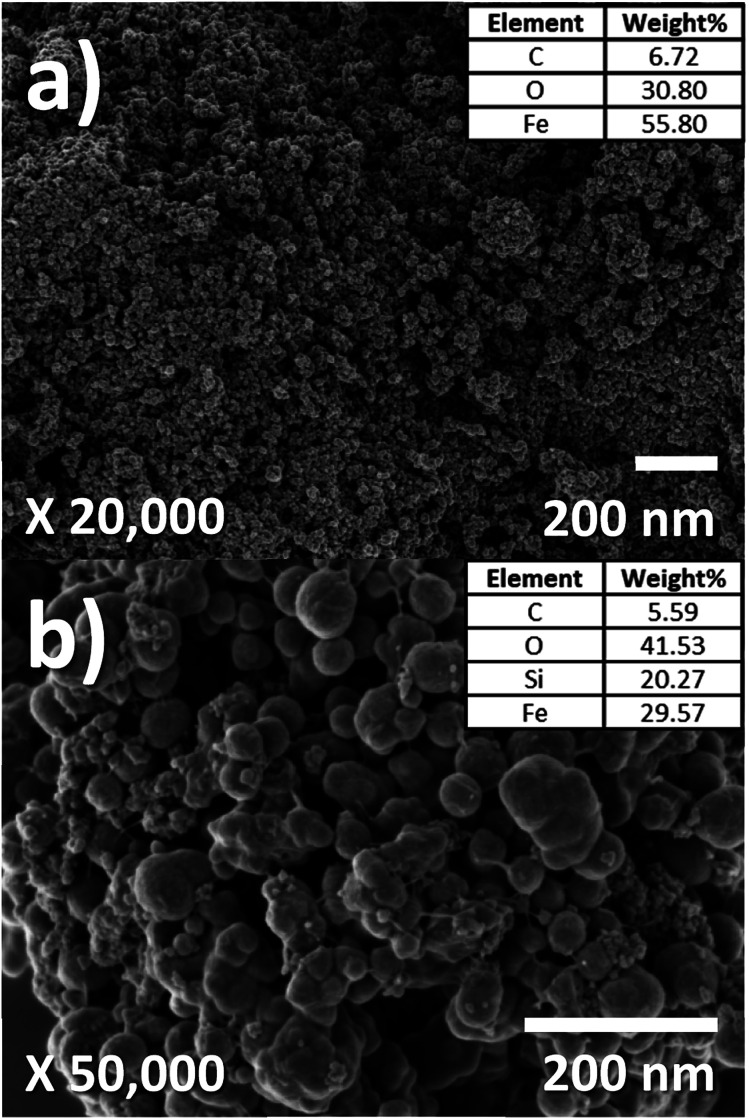
SEM images and EDX analysis of (a) FeO-NPs and (b) FeO@SiO_2_.

SEM images for morphology analysis of FeO-NPs and FeO@SiO_2_–DNA are shown in Fig. 3a and b to complement the TEM analysis. Fig. 3a shows FeO-NPs with an irregular and non-uniform semi-spherical shape, whereas Fig. 3b displays the FeO@SiO_2_–DNA with an increase in particle size attributed to the growth of the SiO_2_ shell. The rougher surface with a spherical shape was observed, as well as an apparent sticking behavior due to the ds-DNA condensation on the surface. Additionally, the elemental composition was acquired using an EDX analysis (see insets in Fig. 3a and b). The content of Fe decreased from 55.80 to 29.57% mainly attributed to the presence of Si atoms (29.6%). Additionally, both samples exhibited high content of C and O, which was ascribed to the presence of C=O, –OH, and Si–O bonds. These results were validated and discussed in the section related to FTIR spectroscopy.

**Fig. 4:**
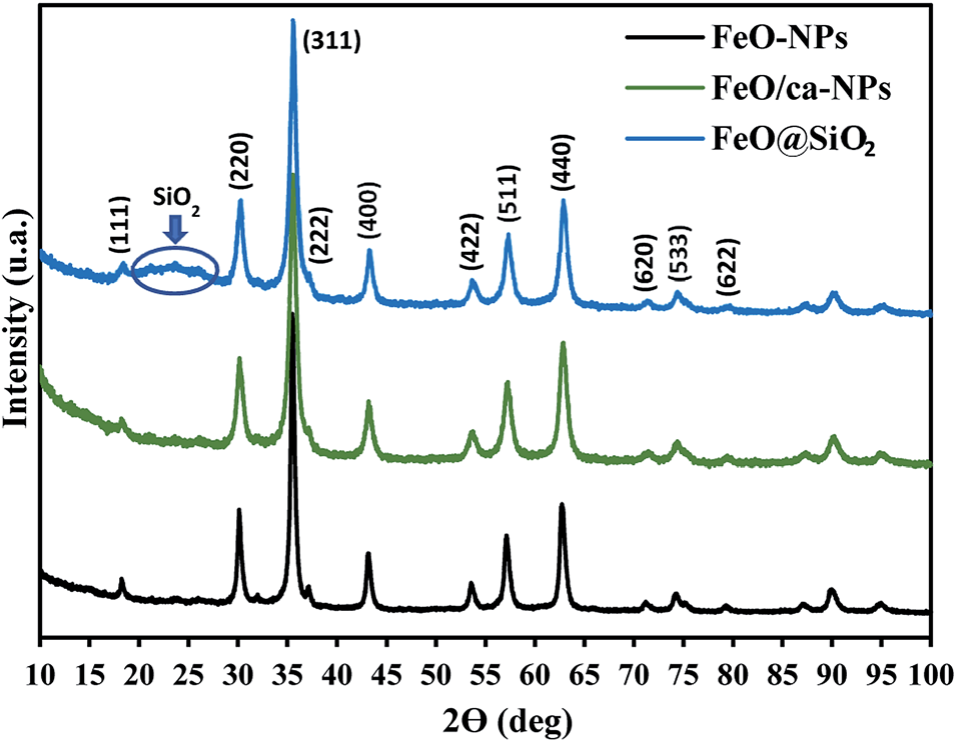
X-ray diffraction (XRD) patterns showing the characteristic peaks and crystalline nature of the magnetic core and the SiO_2_ shell.

The crystal structures of FeO-NPs, FeO/ca-NPs, and FeO@SiO_2_ were evaluated from the XRD patterns in Fig. 4. Characteristic peaks of FeO-NPs are observed at 18, 30, 36, 37, 43, 54, 57, 63, 72, 74, and 79°, which correspond to the crystal planes of (111), (220), (311), (222), (400), (422), (511), (440), (620), (533), and (622), respectively. These peaks are attributed to the face-centered cubic structure of the magnetite phase (Fe_3_O_4_, *Fd*3̄*m*). Moreover, the (400) and (511) planes can be also indexed to the cubic structure of the maghemite phase (γ-F_2_O_3_, *P*4_1_32 space group). This additional indexation suggests the oxidation of the Fe_3_O_4_ phase to produce the structuration of a γ-Fe_2_O_3_ (Fe^2+^-deficient Fe_3_O_4_) phase.^46^ These results are in agreement with the Joint Committee on Powder Diffraction Standards (JCPDS) with the cards no. 19-0629,^47^ 75-0033,^45^ and 65-3107.^48^ After the incorporation of the SiO_2_ shell, no changes were observed in the characteristic peaks for Fe_3_O_4_ and γ-Fe_2_O_3_ phases. The XRD pattern for FeO@SiO_2_ shows a weak and broad peak between 20 and 28°, which was ascribed to the amorphous structure of the SiO_2_ shell.^26^ Additionally, strong and sharp peaks at 36° indicate the crystalline nature, which the average crystallite size was calculated using the Debye–Scherrer method based in eqn (8).^49^8
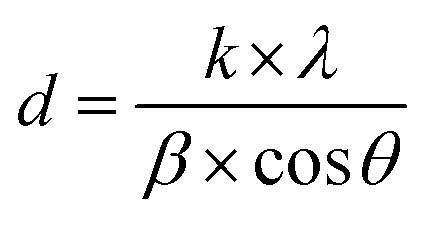
where *d* is the crystallite size (nm), *k* is the Scherrer constant (0.94), *λ* is the wavelength of the X-ray source (0.15405 nm), *β* is the full width at half maximum (FWHM) in radians, and *θ* is the half diffraction Bragg angle. The calculated crystallite sizes were 18.85, 11.68, and 13.38 nm for FeO-NPs, FeO/ca-NPs, and FeO@SiO_2_, respectively. The reduction in the crystallite sizes is attributed to the good dispersion of FeO/ca-NPs, which was previously observed in the TEM images.

**Fig. 5:**
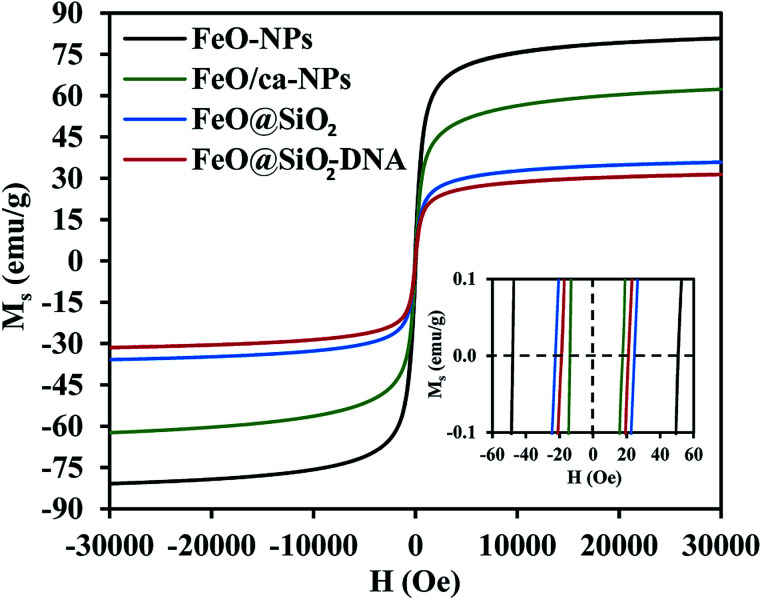
Magnetization *vs.* field curves (*M*_s_*vs. H*) at 300 K to determine changes in the magnetic saturation.

The magnetic nature of the FeO-NPs, FeO/ca-NPs, FeO@SiO_2_, and FeO@SiO_2_–DNA was evaluated through the magnetic hysteresis loops shown in Fig. 5. Here, the magnetization curves suggest a strong magnetic response typical for superparamagnetic materials. The superparamagnetic behavior was confirmed due to the high saturation magnetization (*M*_s_) values reported in Table 1. However, a decrease in *M*_s_ was evidenced and attributed to the presence of the citric acid coating, SiO_2_ shell, and ds-DNA loading. The decrease of the magnetic response was also ascribed to a particle size reduction,^50^ since the citric acid coating promoted a good dispersion of FeO/ca-NPs by breaking the dipolar–dipolar interactions of spins and reducing their coordination.^44^ Additionally, the inset in Fig. 5 shows a low exchange bias (*H*_EB_) and coercive (*H*_C_) fields, which were calculated according to eqn (9) and (10).^51^9
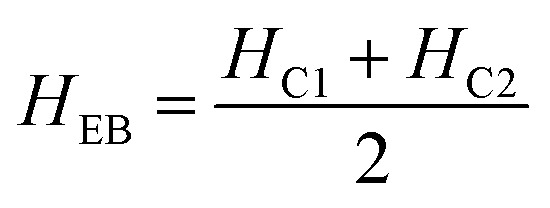
10
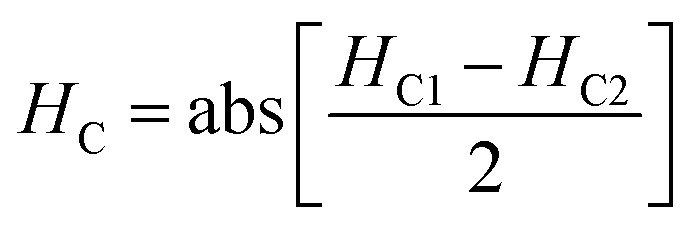
where *H*_C1_ and *H*_C2_ are the left and right coercive fields (Oe). The results of *H*_EB_ and *H*_C_ are shown in Table 1. These results determine the ability to respond to an external magnetic field without delays, becoming demagnetized, or leaving residual magnetism.^52^ Moreover, the absence of an anisotropy surface was proven, and although the *M*_s_ was reduced after modifications, the superparamagnetic nature is still predominant.^44^ Similar core–shell nanostructures exhibited analog superparamagnetic behavior,^53,54^ suggesting that FeO@SiO_2_–DNA are suitable to be recovered and recycled for environmental applications.

**Table 1:** Calculated values of exchange bias (*H*_EB_) and coercive (*H*_C_) fields

Sample	*M*_s_ (emu g^−1^)	*H*_EB_ (Oe)	*H*_C_ (Oe)
FeO-NPs	80.9	1.3	49.4
FeO/ca-NPs	62.4	2.0	15.7
FeO@SiO_2_	35.9	0.8	23.3
FeO@SiO_2_–DNA	31.5	1.1	20.2

**Fig. 6:**
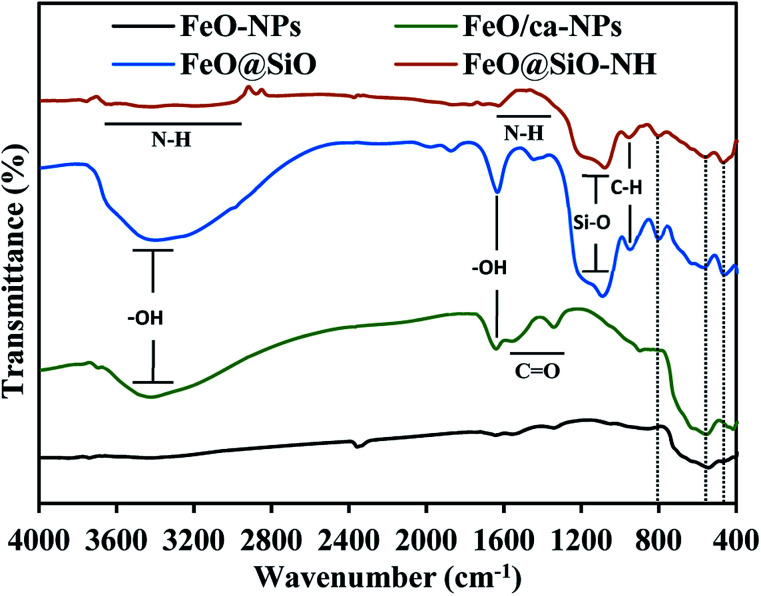
Fourier transform infrared (FTIR) spectra to identify the functional groups content.

The functional groups were identified using the FTIR spectroscopy and showed in Fig. 6. The Fe–O stretching vibration was proved with a broad peak between 509 and 584 cm^−1^, which is the characteristic band for Fe_3_O_4_ and γ-Fe_2_O_3_ phases.^55^ The citric acid coating was confirmed with the appearance of carbonyl (C=O) and hydroxyl (–OH) bonds corresponding to the –COOH group. The –OH stretching vibrations were identified with a broad peak between 3600 and 2700 cm^−1^, and the peak at 1635 cm^−1^. In the case of the C=O bond, peaks at 1547 and 1386 cm^−1^ appeared for the asymmetrical and symmetrical stretching vibrations, respectively. All these peaks confirmed the binding of –COOH groups with iron atoms contained in the FeO-NPs' structure. These bonds promoted the formation of complexes with iron atoms during the *in situ* co-precipitation reaction, leading to the growth of the citric acid layer.^56^ On the other hand, the FeO@SiO_2_ spectrum showed a broader band between 3600 and 2700 cm^−1^, and a more intense peak at 1628 cm^−1^, attributed to a major contribution of –OH groups due to the presence of Si–OH bonds in the SiO_2_ shell. Peaks at 1080, 795, and 455 cm^−1^ were assigned to the asymmetric, symmetric, and bending of Si–O–Si vibrations, respectively, which confirm the successful growth of the SiO_2_ shell.^57^ A shoulder at 625 cm^−1^ indicated the formation of Fe–O–Si complexes through chemical binding between the FeO/ca-NPs and the SiO_2_ shell.^26^ In the case of FeO@SiO_2_–NH_2_, the broadband with low intensity between 3680 and 2916 cm^−1^,^58^ and the shoulder from 1335 to 1612 cm^−1^,^59^ were ascribed to the overlapping of the –OH bands by N–H bonds. The presence of amine groups on the surface was proved, suggesting a suitable surface for ds-DNA condensation. Additionally, a small band near 949 cm^−1^ was identified for the C–H stretching vibration, as a result of remained CH_2_ bindings from the TEOS precursor.

### 3.2. Adsorption efficiency, kinetics, and desorption

FeO@SiO_2_–DNA were used in batch experiments to evaluate the adsorption efficiency of heavy metal ions, including Pb(ii), As(iii), and Hg(ii). The ds-DNA molecules are elongated and random chains with phosphate groups along with the double-stranded coils, which provide a highly negative charged environment. Hence, ds-DNA chains possess the flexibility to form bindings with the positive charges of FeO@SiO_2_–NH_2_, due to the predominant ionic strength interactions with the amine groups.^60^ Accordingly, the type and speed of ds-DNA condensation onto FeO@SiO_2_–NH_2_ depend on the ionic strength of the solution. The ionic solution was a mixture of PEG and NaCl to promote a magnitude volume reduction of the ds-DNA chains to a more compact orientation. The compaction reduces the volume that the chain occupies on the nanostructure's surface, which allows a higher condensation of ds-DNA chains. In this process, the highly charged environment promotes a double electrostatic interaction of the ds-DNA, with the PEG/NaCl mixture and FeO@SiO_2_–NH_2_.^61^ The ds-DNA loading onto FeO@SiO_2_–NH_2_ was determined through the calculation of the adsorption efficiency using the supernatant after the condensation procedure. The adsorption efficiency was around 66% considering a concentration of 100 μg mL^−1^ and 10 mg mL^−1^ of ds-DNA and FeO@SiO_2_–NH_2_, respectively. The efficiency result is directly related to factors such as particle size, which a major ds-DNA loading can be achieved with a higher concentration of FeO@SiO_2_–NH_2_.^33,62^ However, the rapid condensation allowed to achieve a relatively high adsorption efficiency, suggesting high availability of binding sites due to the presence of –NH_2_ groups.

**Fig. 7:**
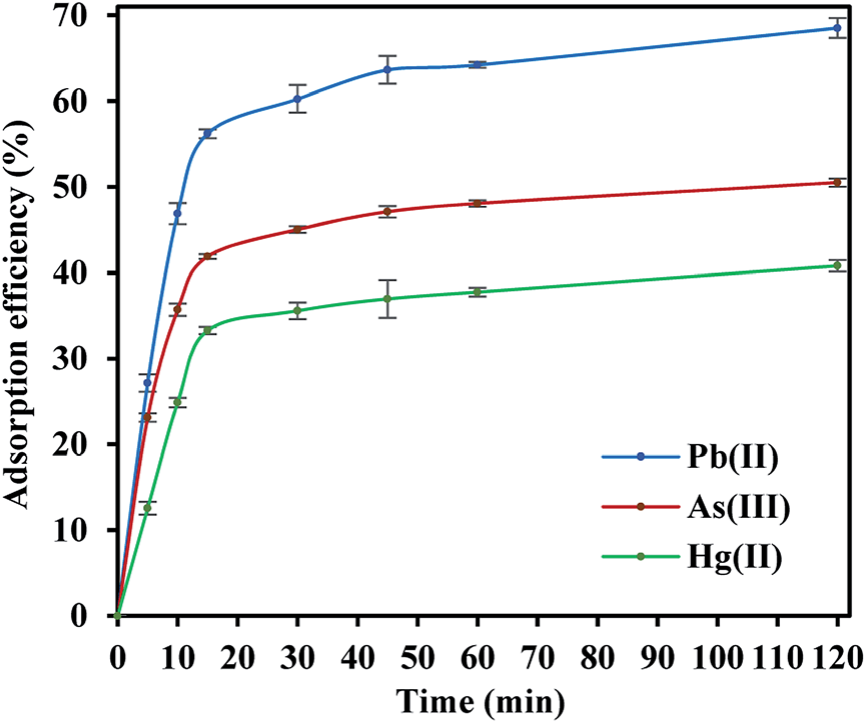
Adsorption efficiency of the FeO@SiO_2_–DNA for the removal of Pb(ii), As(iii), and Hg(ii).

The adsorption efficiencies of FeO@SiO_2_–DNA towards the removal of Pb(ii), As(iii) and Hg(ii) are observed in Fig. 7. The adsorption consisted of three stages for all the metal ions, in which the curves evidence rapid adsorption in the first 15 min. The resulting adsorption rates were attributed to the large number of available active sites offered by the surface of the FeO@SiO_2_–DNA (supported by a total BET surface area of 41.27 m^2^ g^−1^). The adsorption rate slightly decreased in the next 30 min, and then, achieved an equilibrium state after 45 min which was established as the equilibrium time. Additionally, the adsorption efficiency of the FeO@SiO_2_–DNA was different for each metal ions. The adsorption efficiency was 68.51, 50.51, and 40.80% for Pb(ii), As(iii), and Hg(ii), respectively. The differences are attributed to parameters such as the atomic radius (AR), the ratio of AR respect to atomic weight, and the electronegativity, promoting a high selectivity of the FeO@SiO_2_–DNA towards Pb(ii) ions.^63^ These results are following the Hard and Soft Acids and Bases (HSAB) theory, which establishes that Pb(ii) and As(iii) present an intermediate and soft acid nature, respectively.^64^ In the case of Hg(ii), the acid nature is strong enough to lead weak polarization and low stability on the surface of FeO@SiO_2_–DNA.^18^ The ds-DNA contains nucleobases with intermediate and soft acid natures, providing higher selectivity and affinity towards Pb(ii) and As(iii) due to the ionic strength.^65^

The adsorption mechanism was determined by comparing the experimental data to the pseudo-first order, pseudo-second order, Elovich, and intraparticle diffusion models. The parameters were calculated and shown in Table 2, which the coefficient of determination (*R*^2^) indicates the model that better fits. Pseudo-second order was the most suitable model for the experimental data (*R*^2^ above 0.99), with *q*_e_ values of 17.95, 13.16, and 10.89 mg g^−1^ for Pb(ii), As(iii) and Hg(ii), respectively. The *q*_e_ values were similar to those calculated at the equilibrium time and agreed with the adsorption efficiencies displayed in Fig. 7. Additionally, the low values of *k*_2_ suggests the rapid adsorption of metal ions at the beginning. Afterward, the adsorption is reduced and the equilibrium state is achieved due to the decrease in the number of available active sites on the FeO@SiO_2_–DNA.^66^ In the case of the pseudo-first order model, the experimental data showed poor-fitting with *R*^2^ values below 0.6268, and the theoretical adsorption capacities differed from those calculated experimentally. These results suggest that the rate-controlling step and the adsorption mechanism are described by the pseudo-second order model as chemisorption.^67^ Chemisorption involves the electrostatic interaction by sharing electrons between the metal ion, and the positively charged nucleobases contained in the FeO@SiO_2_–DNA.^68^ The electrostatic interaction promotes the formation of a complex surface due to the metal ion is adsorbed chemically, rather than reversible, physical, or mass transfer adsorptions.^69^ Additionally, the experimental data partially followed the Elovich equation model, with *R*^2^ values above 0.84. The Elovich model also predicts chemisorption as the adsorption mechanism, in which an exponential increase of the adsorption is promoted by the availability and heterogenicity of the active sites.^40^ Here, the different activation energies of the heterogeneous sites follow second-order reactions, suggesting a direct relation with the pseudo-second order model.^70^ The rapid and exponential adsorption rate was corroborated with the high *α* values, and were in agreement with the pseudo-second order constant.^71^

**Table 2:** Kinetic model parameters to determine the adsorption mechanism of the FeO@SiO_2_–DNA

Kinetic model	Parameters	Heavy metal ion		
		Pb(ii)	As(iii)	Hg(ii)
	*q*_e,exp_ (mg g^−1^)	17.13	12.63	10.20
Pseudo-first order	*k*_1_ (min^−1^)	0.018	0.015	0.014
	*q*_e_ (mg g^−1^)	4.91	3.07	3.11
	*R* ^2^	0.63	0.47	0.47
Pseudo-second order	*k*_2_ (mg g^−1^ min^−1^)	0.009	0.015	0.011
	*q*_e_ (mg g^−1^)	17.95	13.16	10.89
	*R* ^2^	0.99	0.99	0.99
Elovich	*β* (g mg^−1^)	0.34	0.50	0.49
	*α* (mg g^−1^ min^−1^)	12.01	14.04	3.84
	*R* ^2^	0.85	0.87	0.84
Intraparticle diffusion	*k*_p_ (mg g^−1^ min^−1/2^)	0.97	0.66	0.67
	*C* (mg g^−1^)	8.25	6.65	4.11
	*R* ^2^	0.68	0.70	0.67

**Fig. 8:**
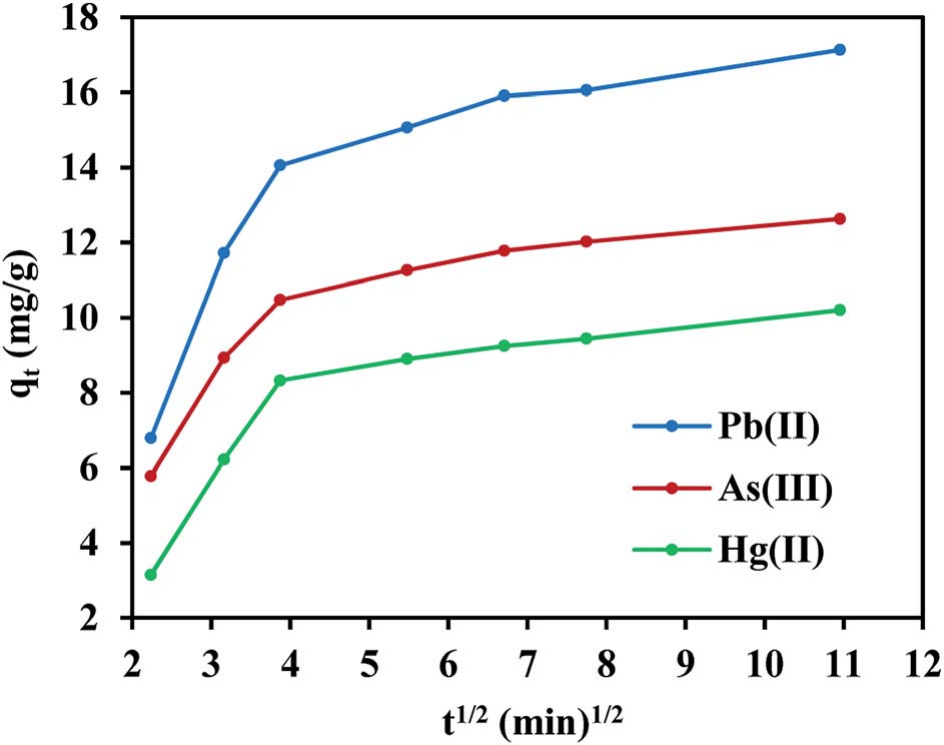
Two-stage adsorption mechanism established by the intraparticle diffusion model.

On the other hand, the experimental data does not fit the intraparticle diffusion model (with *R*^2^ below 0.70), indicating the absence of transport by diffusion through the pores of the FeO@SiO_2_–DNA. In such cases typically three main stages are expected, (i) large pore diffusion or boundary layer diffusion, (ii) micropore diffusion, and (iii) equilibrium adsorption.^68^ In this study, a two-stage process for all three metal ions was observed in Fig. 8. The first stage was attributed to the electrostatic interaction on the boundary layer, leading to rapid adsorption due to the presence of a large number of active sites. The rapid adsorption on the boundary layer was proportional to the *C* values, which were 8.25, 6.65 and 4.11 mg g^−1^ for Pb(ii), As(iii) and Hg(ii), respectively. Additionally, the high adsorption rate on the boundary layer was in accordance with the BJH analysis (low pore volume and pore size of 0.23 cm^3^ g^−1^ and 19.92 nm, respectively). The structure with low porosity confirmed the absence of a second stage due to the saturation of the surface and the low pore diffusion. Finally, the rapid adsorption decreased and the equilibrium state was achieved, which indicates that the adsorption process was mainly through boundary layer diffusion. These results suggest the FeO@SiO_2_–DNA as a suitable nanoadsorbent for heavy metal ions removal in aqueous solution, compared to similar core–shell nanostructures reported in the literature.^30,69^

**Fig. 9:**
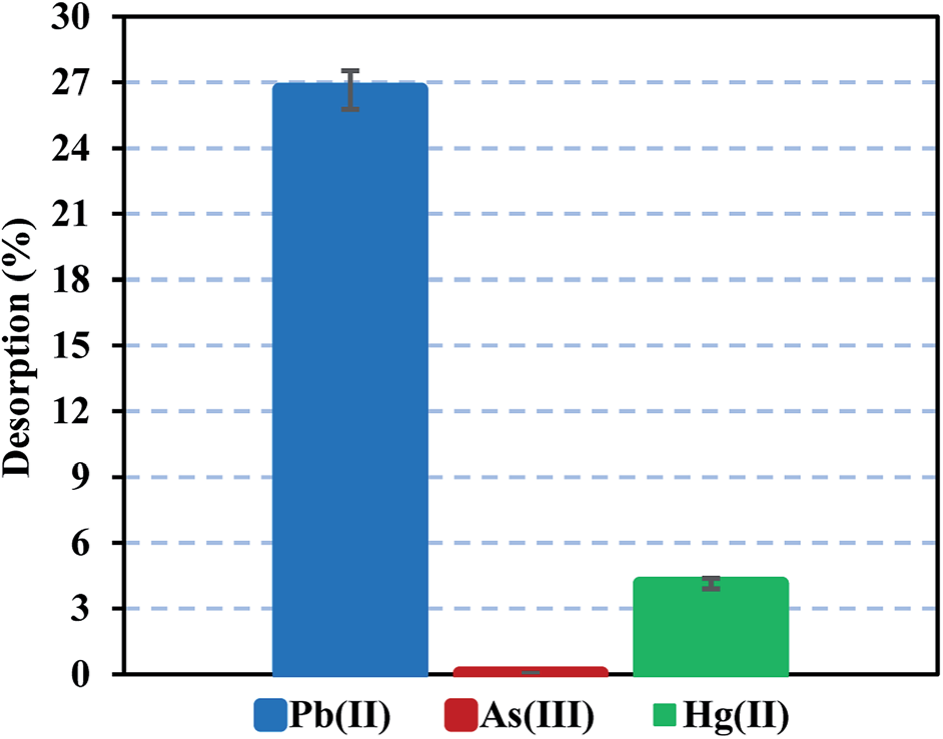
Desorption performance of Pb(ii) and Hg(ii) using EDTA, and desorption of As(iii) using NaOH.

Heavy metal elution allows determining the applicability and stability of the FeO@SiO_2_–DNA. Therefore, desorption experiments were performed using two different eluent agents, EDTA for desorption of Pb(ii) and Hg(ii), and NaOH for desorption of As(iii). EDTA is widely investigated to recover various heavy metal ions through the formation of chelates. The chelation contributes to an easy migration of heavy metal ions from the surface of the FeO@SiO_2_–DNA to an aqueous solution.^72^ In the case of NaOH, a strong base promotes an electrostatic interaction between the –OH groups and the cationic nature of the metal ion. The –OH groups displace the metal ions from the surface of the FeO@SiO_2_–DNA, as well as the Na cations weaken the electrostatic interaction.^73^Fig. 9 shows low desorption rates for each metal ion considering the use of strong eluent agents. The poor affinity to the effluent agents was attributed to strong electrostatic interactions between the cationic surfaces of the metal ion and the available active sites of the FeO@SiO_2_–DNA.^74^ The strong electrostatic interaction promotes constant and large stability of the metal ion on the surface of FeO@SiO_2_–DNA.^75^ The stability is also attributed to the ds-DNA chains, which provide high affinity through a large number of functional groups interacting with the cationic metal ions.^65^ These results suggest that the heavy metal ions remain stable on the surface of the FeO@SiO_2_–DNA, avoiding desorption in the eluent agents seeking their migration.

## 4. Conclusions

This study reports the synthesis of core–shell nanostructures composed of a functionalized magnetic core and a SiO_2_ shell. The carboxylic acids coating the FeO-NPs improved the stability for better dispersion, and promoted the growth of uniform and dispersed FeO@SiO_2_. The rapid loading of ds-DNA was ascribed to the large number of available active sites (–NH_2_ groups) on the cation surface of the FeO@SiO_2_. No changes in the crystal structure were observed after the modification of FeO-NPs, and excellent coordination of spins in the magnetic core confirmed the superparamagnetic nature. The magnetic response in all samples was attributed to the good dispersion and stability conferred by the citric acid coating.

The adsorption efficiency of the FeO@SiO_2_–DNA was evaluated for Pb(ii), As(iii), and Hg(ii) removal. Pseudo-second order and Elovich models fitted the experimental data indicating heterogeneous chemisorption based on electrostatic interaction. The selectivity is related to the similar acid nature of the metal ion with the nucleobases in the ds-DNA, highlighting the high ionic strength for rapid adsorption and the good stability on the surface. The FeO@SiO_2_–DNA represent a suitable and promising nanostructure for remediation of water sources through rapid adsorption techniques. Future works can be addressed to increase the adsorption efficiency by enhancing the ds-DNA loading percentage for more active sites, including experiments using single-stranded chains. The low desorption was attributed to the strong interaction and stability. However, the concentrations of the eluent agents and the operating conditions need to be optimized to reduce the interaction and to regenerate the nanostructures.

## Conflicts of interest

There are no conflicts to declare.

## Supplementary Material

RA-010-D0RA06743A-s001
