# Supplementary material for: Synthesis of FeO@SiO2–DNA core–shell engineered nanostructures for rapid adsorption of heavy metals in aqueous solutions
Source: RSC Adv. 2020 Oct 27;10(64):39284–94. doi: 10.1039/d0ra06743a (PMC9057351; doi:10.1039/d0ra06743a)
Supplement: RA-010-D0RA06743A-s001 [file RA-010-D0RA06743A-s001.pdf]

Electronic Supplementary Material (ESI) for RSC Advances.
This journal is © The Royal Society of Chemistry 2020

SUPLEMMENTARY MATERIAL

Synthesis of FeO@SiO_2-DNA core-shell engineered nanostructures for rapid adsorption of heavy metals in aqueous solutions

David Patiño-Ruiz[a], Lars Rehmann[b], Mehrab Mehrvar[c], Edgar Quiñones-Bolaños[a,d], and Adriana Herrera[a,e]*

[a]Engineering Doctorate Program, Nanomaterials and Computer-Aided Process Engineering Research Group, Universidad de Cartagena, Cartagena, 130010, Colombia

[b]Department of Chemical and Biochemical Engineering, University of Western Ontario, London, N6A 3K7, Canada

[c]Department of Chemical Engineering, Ryerson University, Toronto, M5B 2K3, Canada

[d]Civil Engineering Program, Environmental Modelling Research Group, Universidad de Cartagena, Cartagena, 130010, Colombia

[e]Chemical Engineering Program, Nanomaterials and Computer-Aided Process Engineering Research Group, Universidad de Cartagena, Cartagena, 130010, Colombia

*Email: aherrerab2@unicartagena.edu.co

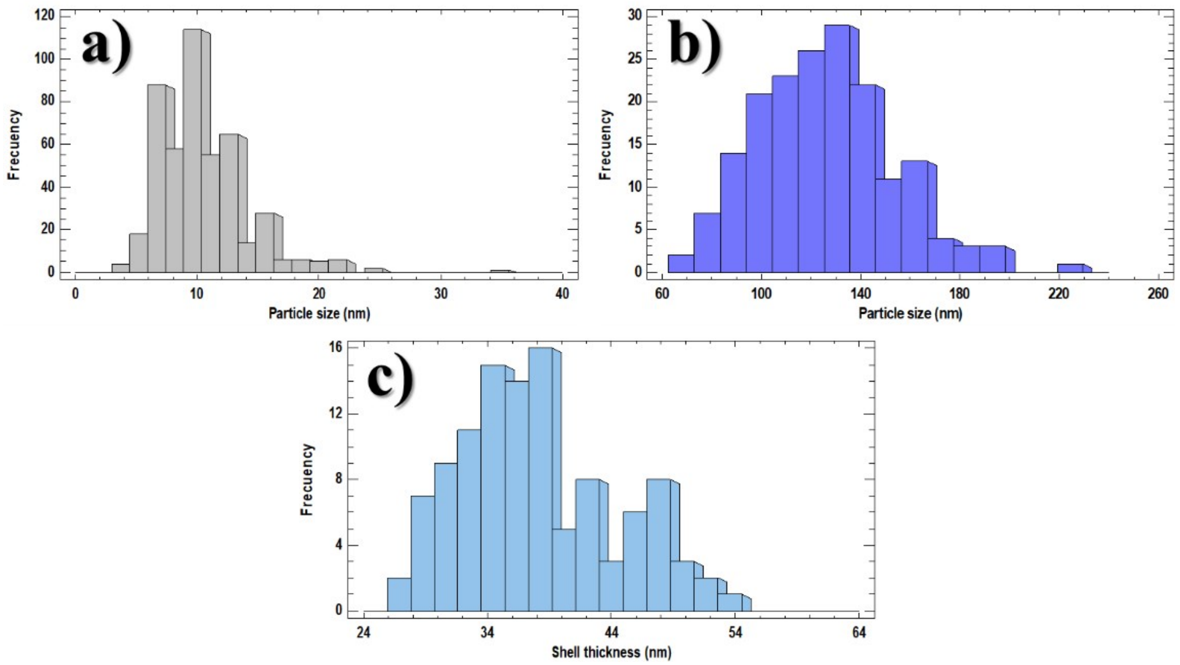

Figure S1. Particle size distribution of **(a)** FeO/ca-NPs (10.2 ± 3.7 nm), **(b)** FeO@SiO_2 (125 ± 27 nm), and **(c)** SiO_2 shell thickness (38 ± 6 nm).

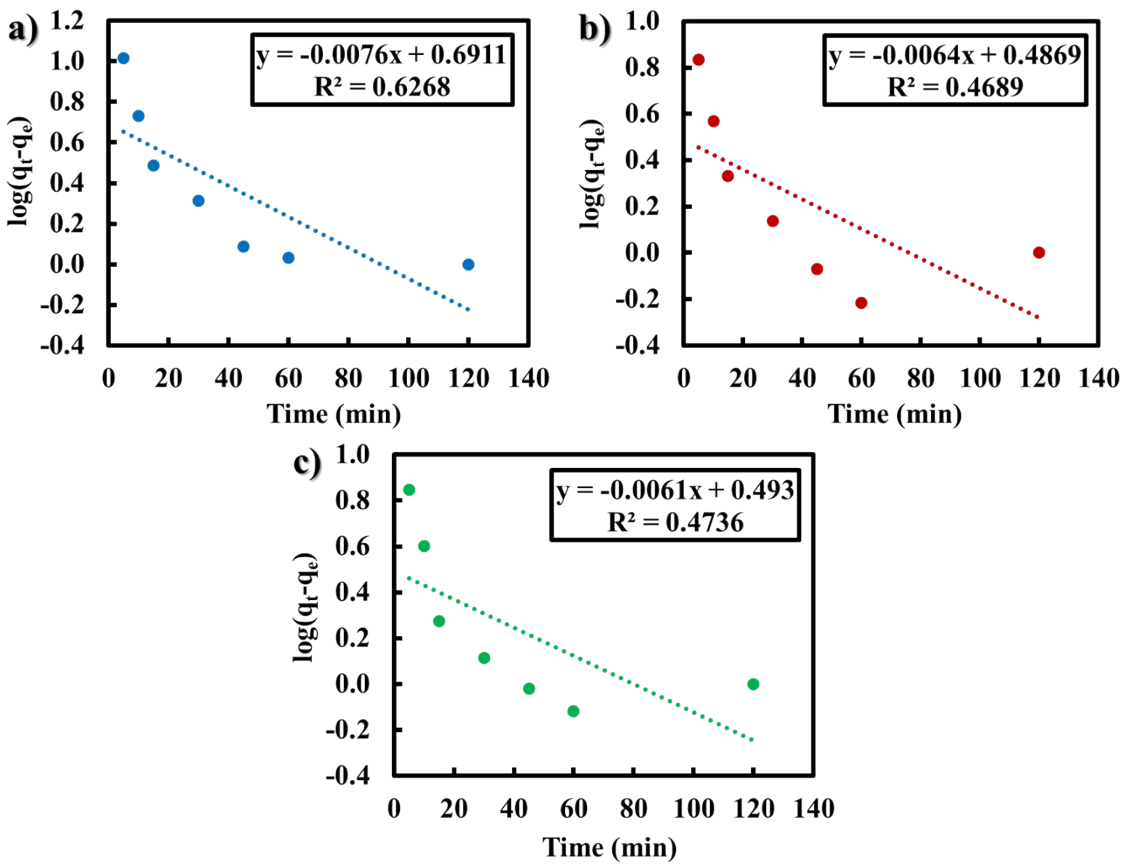

Figure S2. Experimental data fitting based on the Pseudo-first order model for the adsorption of **(a)** Pb(II), **(b)** As(III), and **(c)** Hg(II).

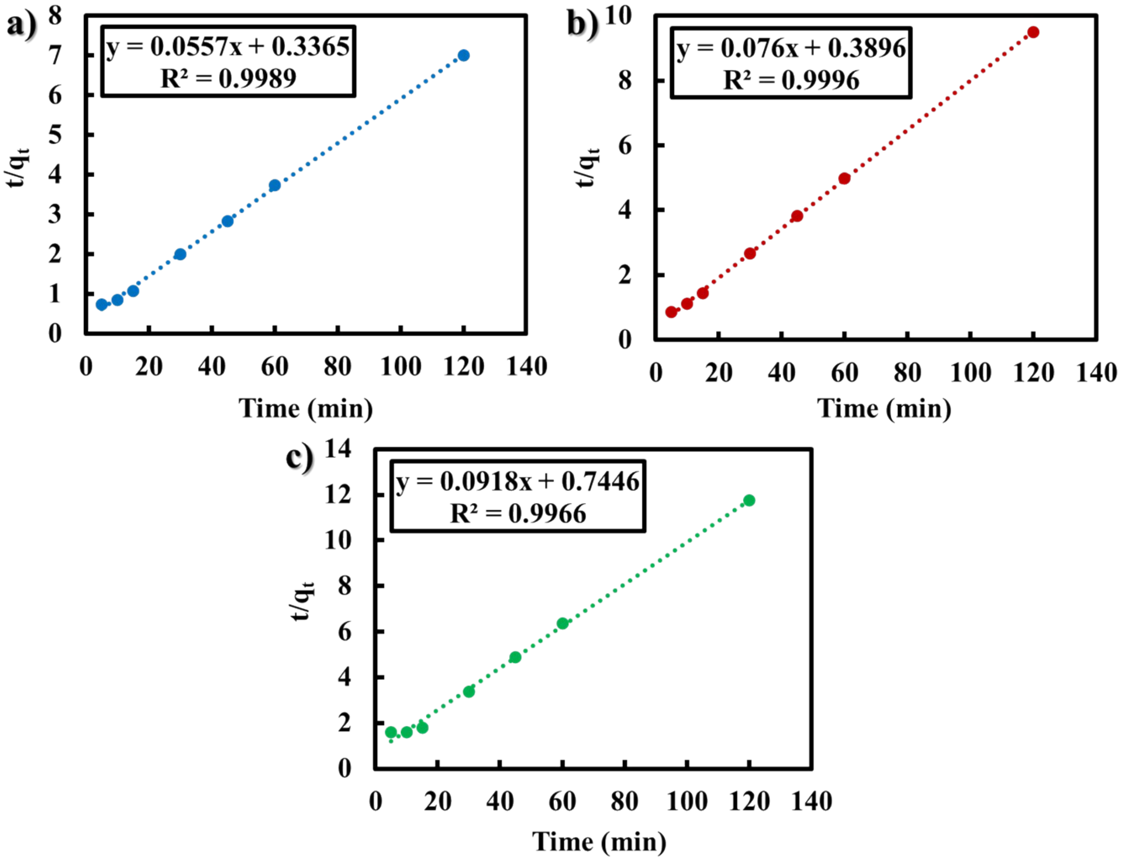

Figure S3. Experimental data fitting based on the Pseudo-second order model for the adsorption of **(a)** Pb(II), **(b)** As(III), and **(c)** Hg(II).

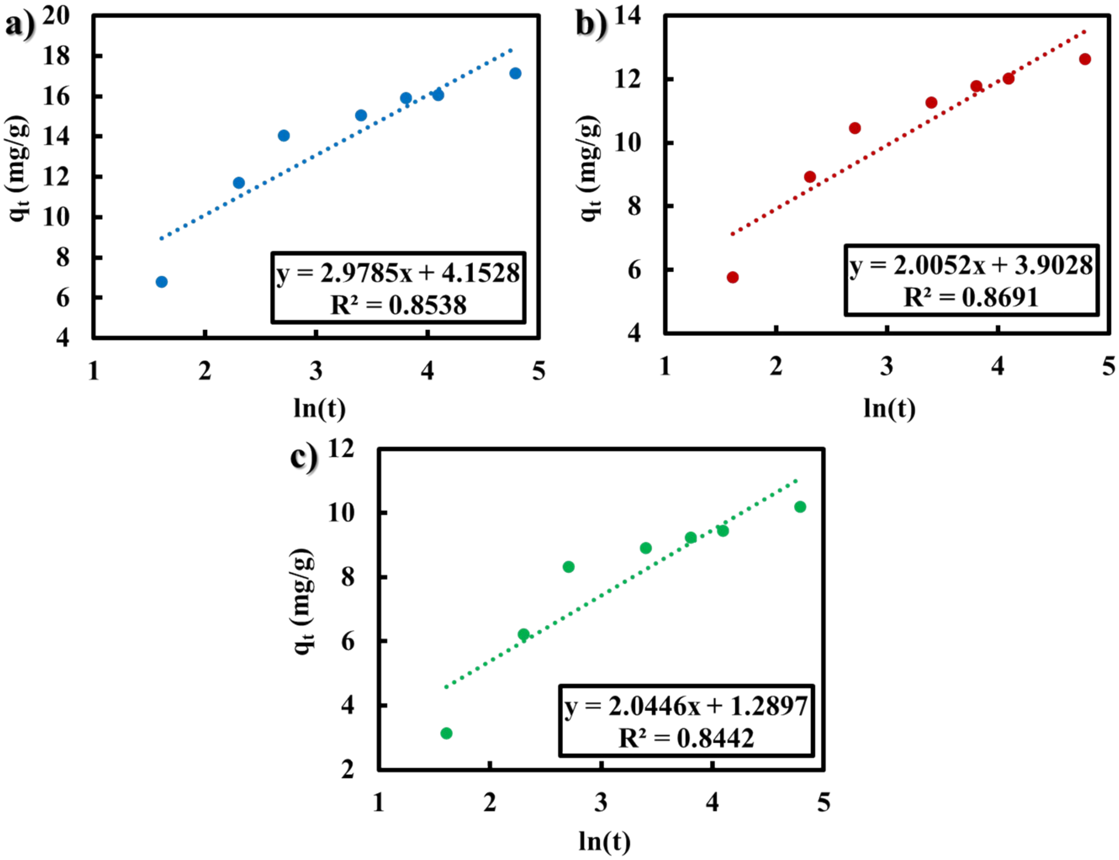

Figure S4. Experimental data fitting based on the Elovich model for the adsorption of **(a)** Pb(II), **(b)** As(III), and **(c)** Hg(II).
